# Time-resolved characterization of the innate immune response in the respiratory epithelium of human, porcine, and bovine during influenza virus infection

**DOI:** 10.3389/fimmu.2022.970325

**Published:** 2022-08-19

**Authors:** Laura Laloli, Manon Flore Licheri, Lukas Probst, Matthias Licheri, Mitra Gultom, Melle Holwerda, Philip V’kovski, Ronald Dijkman

**Affiliations:** ^1^ Institute for Infectious Diseases, University of Bern, Bern, Switzerland; ^2^ Graduate School for Cellular and Biomedical Sciences, University of Bern, Bern, Switzerland; ^3^ Institute of Virology and Immunology (IVI), Bern, Switzerland; ^4^ Department of Infectious Diseases and Pathobiology, Vetsuisse Faculty, University of Bern, Bern, Switzerland

**Keywords:** Influenza C virus, influenza D virus, zoonoses, cross-species transmission, innate immune response, respiratory epithelium, virus – host interactions

## Abstract

Viral cross-species transmission is recognized to be a major threat to both human and animal health, however detailed information on determinants underlying virus host tropism and susceptibility is missing. Influenza C and D viruses (ICV, IDV) are two respiratory viruses that share up to 50% genetic similarity, and both employ 9-O-acetylated sialic acids to enter a host cell. While ICV infections are mainly restricted to humans, IDV possesses a much broader host tropism and has shown to have a zoonotic potential. This suggests that additional virus–host interactions play an important role in the distinct host spectrum of ICV and IDV. In this study, we aimed to characterize the innate immune response of the respiratory epithelium of biologically relevant host species during influenza virus infection to identify possible determinants involved in viral cross-species transmission. To this end, we performed a detailed characterization of ICV and IDV infection in primary airway epithelial cell (AEC) cultures from human, porcine, and bovine origin. We monitored virus replication kinetics, cellular and host tropism, as well as the host transcriptional response over time at distinct ambient temperatures. We observed that both ICV and IDV predominantly infect ciliated cells, independently from host and temperature. Interestingly, temperature had a profound influence on ICV replication in both porcine and bovine AEC cultures, while IDV replicated efficiently irrespective of temperature and host. Detailed time-resolved transcriptome analysis revealed both species-specific and species uniform host responses and highlighted 34 innate immune-related genes with clear virus-specific and temperature-dependent profiles. These data provide the first comprehensive insights into important common and species-specific virus-host dynamics underlying the distinct host tropism of ICV and IDV, as well as possible determinants involved in viral cross-species transmission.

## Introduction

Cross-species, or interspecies, transmission of viruses is the most significant cause of disease emergence in animals and humans, with often severe consequences to both animal and human health (1, 2). The viral determinants affecting interspecies transmission and pathogenesis of many respiratory viruses, including influenza viruses, are only partially understood, and there is still a great gap in our knowledge on cellular effectors and their mechanisms in the airway epithelium among different host species (3). One of the main reasons is the lack of appropriate experimental models to characterize host restriction determinants, and corresponding viral evasion strategies in molecular detail (4).

Influenza C virus (ICV) and influenza D virus (IDV) are enveloped RNA viruses belonging to the *Orthomyxoviridae* family. While ICV and IDV belong to different genera, Gammainfluenzavirus and Deltainfluenzavirus, respectively, these viruses are phylogenetically more closely related to each other than to the more well-known influenza viruses of the Alphainfluenzavirus and Betainfluenzavirus genera (5). Moreover, both ICV and IDV have 7 genomic segments sharing up to 50% overall genetic similarity and employ the Hemagglutinin-Esterase-Fusion (HEF) glycoprotein for binding to 9-O-acetylated sialic acids (9-O-SA) for host cell entry (6, 7). Interestingly, both the ICV and IDV HEF glycoproteins can bind to the luminal surface of human, porcine, and bovine respiratory epithelium, although both viruses noticeably differ in their respective host tropism (7). ICV is predominantly a human pathogen, causing respiratory diseases in infants and children, and has sporadically been associated with respiratory infections in swine and cattle (8–11). In contrast, IDV exhibits an intrinsic broad host tropism among different livestock (12–15), with cattle as the main host reservoir (12, 16–18), and was shown to have a zoonotic potential (19–24). These studies suggest that factors other than receptor usage might play a role in the distinct host tropism between ICV and IDV.

For successful viral interspecies transmission, several barriers must be overcome in a new host species. These barriers can be categorized into three major stages (i) viral entry through availability of the cellular receptor and proteases, (ii) viral replication and efficiency to antagonize the cell-intrinsic innate immune response followed by (iii) viral egress and release of infectious progeny virus (1, 25). As the respiratory tract is the primary entry port for respiratory viruses, the targeted airway epithelial cell populations fulfil an important barrier role in restricting viral replication through modulating the cell-intrinsic innate immune response (26). We and others have recently shown that temperature can influence both viral replication efficiency and the amplitude of the cell-intrinsic innate immune response (27, 28). However, to which extent these factors also influence interspecies transmission of respiratory viruses currently remains elusive.

In this study we established biologically relevant well-differentiated primary airway epithelial cell (AEC) cultures derived from human, porcine, and bovine origin to investigate the influence of temperature on viral replication kinetics and host innate immune response dynamics during ICV and IDV interspecies transmission. This analysis revealed that temperature strongly affects ICV replication in porcine and bovine AEC cultures, whereas IDV replicated efficiently independent from temperature and host species. Moreover, despite these marked differences, both ICV and IDV predominantly infect ciliated cells, irrespective of host species and temperature. These results indicate that factors other than cell tropism, such as different temperature and host species can influence replication of influenza viruses. Interestingly, our time-resolved transcriptome analysis uncovered both species-specific and common host responses and highlighted 34 innate immune-related genes with clear virus-specific and temperature-dependent profiles. We observed that ICV replication efficiency, but not IDV, inversely correlated with the magnitude of the innate immune response dynamics in porcine and bovine AEC cultures. Combined this knowledge provides first insights into the distinctive replication kinetics and host tropism of ICV and IDV, as well as a detailed characterization of the innate immune response of the airway epithelium of species relevant to the host range of both ICV and IDV.

## Materials and methods

### Cell line

The human rectal tumor 18G cell line (HRT-18G; ATCC) was maintained in Dulbecco’s minimum essential medium (DMEM; Gibco) supplemented with 5% heat-inactivated fetal bovine serum (FBS), 100 µg/mL streptomycin and 100 IU/mL penicillin (Gibco) and propagated at 37°C in a humidified incubator with 5% CO_2_. Cell line authenticity was confirmed by Short Tandem Repeat (STR) profiling (Microsynth) and mycoplasma contamination was excluded using the MycoAlert PLUS Mycoplasma Detection Kit according to manufacturer protocol (Lonza).

### Viruses

For virus stock production, both ICV (C/Johannesburg/1/66) and IDV (D/bovine/Oklahoma/660/2013) were propagated on HRT-18G cells for 96 hours at 33°C or 37°C, respectively, using Eagle’s Minimum Essential Medium (MEM) with Glutamax (Gibco), that was additionally supplemented with 0.5% bovine serum albumin (Sigma-Aldrich), 15 mmol/L of HEPES (Gibco), 100 µg/mL streptomycin and 100 IU/mL penicillin (Gibco), and 0.25 µg/mL acetylated trypsin isolated from the bovine pancreas (Sigma-Aldrich). Virus containing supernatant was cleared from cell debris through centrifugation for 5 min at 500x *rcf* before aliquoting and storage at -80°C.

### Primary airway epithelial cell cultures

Human AEC cultures were established from airway cells isolated from tracheobronchial tissue of patients (>18 years old) undergoing pulmonary resection, whereas AEC cultures of swine and bovine origin were established from post-mortem tracheobronchial epithelial tissue. Isolation and culturing of well-differentiated primary airway epithelial cell (AEC) cultures was performed as previously described (29). Briefly, cryopreserved cells were thawed and expanded for 1 week in Bronchial Epithelial cell Growth Medium (BEGM; Gibco), supplemented with various inhibitors to promote cell proliferation and maintaining a basal cell phenotype (30), in a collagen-coated cell culture flask. Upon 80% confluency, cells were seeded into collagen type IV-coated porous inserts in 24-well cluster plates (Corning) in BEGM medium in a liquid-liquid interface. Once cell confluency was reached, cells were airlifted and the basolateral medium was exchanged with species-optimized air-liquid interface (ALI) medium (29, 31). For both human and bovine AEC cultures the concentration of recombinant human epidermal growth factor (rhEGF; Repligen) in the ALI medium was increased to 25ng/ml. Additionally, the bovine ALI medium was supplemented with 2.5µM of the γ-secretase inhibitor DAPT (Tocris), whereas the concentration of the photostable synthetic analogue of all-trans retinoic acid (EC23, Enzo Life Sciences) was increased to 70 nM in only the porcine ALI medium. The basolateral medium was replenished 3 times per week, whereas the apical side was washed with Hank’s Balanced Saline Solution (HBSS; Gibco) once a week. All AEC cultures were allowed to differentiate for 4 weeks at 37˚C in a humidified incubator with 5% CO_2_.

### Infection of AEC cultures of human, porcine, and bovine origin

Well-differentiated AEC cultures were inoculated at the apical side with 10’000 tissue culture infectious dosis 50 (TCID_50_) of either ICV or IDV diluted in HBSS. Following 1-hour incubation at either 33°C or 37°C, virus inoculum was aspirated, and the apical surface was rinsed three times with HBSS, whereby the final wash was collected as the 1-hour post-infection (hpi) time point. The AEC cultures were incubated at the indicated duration and temperatures in a humidified incubator with 5% CO_2_. Production of viral progeny was monitored by incubating 100 μl of HBSS on the apical surface 10 minutes prior to the indicated time points. The collected apical washes were diluted 1:1 with virus transport medium (VTM), and stored at −80˚C for later analysis (29).

### Virus titration by tissue culture infectious dosis 50

One day prior to virus titration, HRT-18G cells were seeded in 96-well cluster plates at a density of 40’000 cells per well. The following day, culture medium was aspirated, cells were washed once with PBS and subsequently overlaid with 50 μL of infection medium (23). Virus stocks or apical washes collected during the viral kinetic experiments were 10-fold serial diluted in infection medium, from which 50 μL were transferred in six technical replicates per sample to 96-well cluster plates. Inoculated HRT-18G cells were incubated for 5 days at 33°C or 37°C for the titration of ICV and IDV, respectively, in a humidified incubator with 5% CO_2_. Due the lack of cytopathogenicity upon infection, 50 μL of supernatant from each reciprocal dilution was mixed with 50 μL of 1% (v/v) chicken red blood cells solution in a V-bottom 96-well cluster plate. The chicken blood was diluted 1:1 in Alsever’s solution (Sigma-Aldrich) and used within three weeks. Following incubation for 30 minutes at room temperature, plates were tilted 45° for 30 seconds to record the presence or absence of virus-induced hemagglutination in each reciprocal dilution. This information was used to calculate the virus titer according to the protocol of Spearman-Kärber (32).

### Immunofluorescence analysis of virus-infected AEC cultures

Well-differentiated AEC cultures were fixed with 4% (v/v) neutral buffered formalin (Formafix) and processed as previously described (29). For the detection of ICV-positive cells, human intravenous immunoglobulins (IVIg; Sanquin) were used. For the detection of IDV-positive cells, AEC cultures were incubated with a custom rabbit polyclonal antibody directed against the NP of the prototypic D/bovine/Oklahoma/660/2013 strain (Genscript). Alexa Fluor^®^ 488-labeled donkey anti-human IgG (H+L) (Jackson immunoresearch) or Alexa Fluor^®^ 488-labeled donkey anti-rabbit IgG (H+L) were used as secondary antibodies for ICV and IDV, respectively. To visualize the cilia and tight junctions the Alexa Fluor 647-conjugated rabbit anti-β-tubulin (9F3; Cell Signaling Technology) and Alexa Fluor 594-conjugated mouse anti-Zonula Occludens 1 (ZO-1) (1A12; Thermo Fisher Scientific) were used. Cell nuclei were visualized using 4’,6-diamidino-2-phenylindole (DAPI; Thermo Fisher Scientific). The immunostained inserts were mounted onto slides with DakoFluorescence Mounting Medium (Agilent), embedded in ProLong™ Diamond Antifade Mountant (Thermo Fisher Scientific) and overlaid with 0.17 mm high-precision coverslips (Marienfeld).

For visual characterization, images from human, porcine, and bovine AEC cultures after 4-6 weeks of differentiation in an air-liquid interface, were acquired with either 4x (0.13 NA) air objective on a Cytation 5 multimode reader (Biotek) and processed and stitched using the Gen5 Image prime software package (v3.08.01) or with 20x (0.4 NA) air objective of EVOS FL Auto 2 Imaging System (Thermo Fisher Scientific). All images were further processed using Fiji Software packages (33). Brightness and contrast were adjusted identically to their corresponding controls. Figures were assembled using the FigureJ plugin (34). For the quantification of ciliated cells and the cell tropism of ICV and IDV, images were acquired with 20x (0.45 NA) air objectives on a Cytation 5 multimode reader (Biotek) for human and porcine AEC cultures, whereas bovine AEC samples were imaged on a DeltaVision Elite High-Resolution imaging system (GE Healthcare Life Sciences equipped with 40x oil immersion objective (1.4 NA), by acquiring 200 to 300 nm z-stacks over the entire thickness of the sample. Images were deconvolved using the integrated softWoRx software.

Segmentation of individual cells in each image using the ZO-1 channel was performed using Cellpose (1.0.0) (35) and scikit-image in Python (version 3). Following segmentation, the mean intensity of both channels corresponding to ICV or IDV and ciliated cell staining was measured for every detected cell. An average of 10^5^ (human), 10^6^ (swine) and 10^4^ (bovine) cells were measured. Infected cells were defined as those with the mean intensity higher than the mean + 3 SD of the corresponding uninfected control cells. Similarly, distinction of ciliated cells from non-ciliated cells was performed by multimodality assessment (36) using R (version 4.1.3).

### Bulk RNA barcoding and sequencing

Total cellular RNA from mock and virus-infected AEC cultures was extracted using the NucleoMag RNA kit (Macherey-Nagel) according to the manufacturer’s guidelines on a Kingfisher Flex Purification system (Thermo Fisher Scientific). Total RNA concentration was quantified with the QuantiFluor RNA System (Promega) according to the manufacturer’s guidelines on a Cytation 5 multimode reader (Biotek). The BRB-seq libraries were generated and sequenced as described previously to a depth of approximately 5 million raw reads per sample (Alithea genomics) (37). The host genome count matrices were generated by aligning the sequencing reads with STAR (v2.7.9a) against the corresponding reference genomes of *Homo sapiens* (GRch38.100), *Sus scrofa* (Sscrofa 11.1.100), or *Bos taurus* (ARS_UCD1.2.100). The viral count matrices were generated by aligning the sequencing reads with STAR (v2.7.9a) against a concatenation of the corresponding reference genomes of ICV (GenBank AF170573 – AF170576, AM410041 – AM410043) and IDV (GenBank KF425659 – KF425665). The resulting host and viral count matrices with unique molecule identifier (UMI) counts were subsequently used as input for data analysis.

### DEseq2

All downstream analyses were performed using R (version 4.1.0). Following low counts filtering per gene (>10 reads in at least 3 samples), library normalization and global expression differences between uninfected and virus-infected samples were quantified for each host species individually using the DESeq2 package (version 1.28) with a fold change (FC) cut-off of ≥ 1.5 and a false discovery rate (FDR) of ≤ 0.05 (38). For the analysis at either 33°C or 37°C, samples were split by temperature prior to differential expression analysis (e.g., subset of all samples for all time points at either 33°C or 37°C), and infected samples were compared to uninfected samples using the design ~ Donor + Condition. In case of the temporal analysis, the samples were kept together, and the identification of significant differentially expressed (DE) genes over time was performed using the likelihood ratio test (LRT) with both the complete design ~ Donor + Condition + TT + Condition TT (TT = conjugation of the Time and Temperature variables) and the reduced design ~ Donor + Condition + TT with a FDR of ≤ 0.01. Hierarchical gene clustering was subsequently performed on a variance-stabilizing transformation (VST) processed count matrix of identified DE genes using the degPatterns function from the DEGreports package (39). Venn diagrams of overlapping DE genes were generated using the ggVennDiagram package (40). Pathway enrichment analysis was performed using the gProfiler2 package for R (41). The 5 most significantly enriched pathways with *p*-value of ≤ 0.05 were visualized using the enrichplot package (version 1.14.2) (42). The merging of DE gene datasets from individual host species was done based on human gene symbol, by converting porcine and bovine orthologous genes to human gene symbols using the gProfiler2 package (41). Further data analysis and visualization was performed using a variety of additional packages in R, including ComplexHeatmap (43) and Tidyverse (44).

### Statistical testing

For assessing normal sample distribution, Shapiro-Wilk normality test (p>0.05) was performed. Statistical significance was analyzed using two-way ANOVA for viral replication kinetics and paired pairwise t-tests for the other analyses (significance legend: *p < 0.05, **p < 0.01, ***p < 0.001). Analyses were performed using R (version 4.1.3).

## Results

### Distinct replication kinetics for ICV and IDV in AEC cultures of human, porcine, and bovine origin

**Figure 1:**
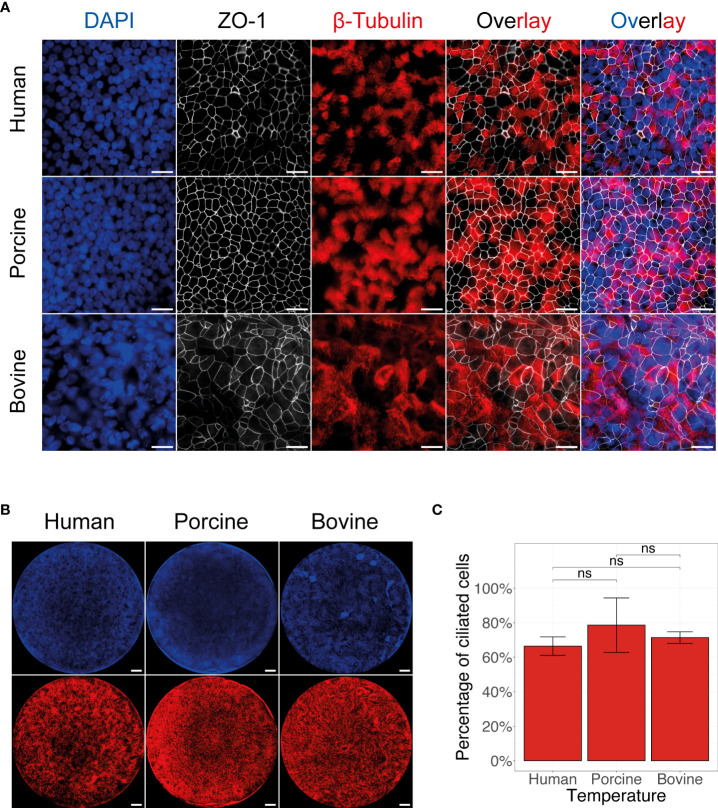
Morphological overview of human, porcine, and bovine AEC cultures. Representative fluorescence images of human, porcine, and bovine AEC cultures revealing the distribution of the cell nuclei (DAPI, blue), tight junctions (ZO-1, white), and cilia (β-tubulin, red). Scale bar: 20 µm **(A)** and 500 µm **(B)**. Quantification of the mean percentage of ciliated cells present in 4 to 6-week-old well-differentiated AEC cultures of human, porcine, and bovine origin. Data represent the mean percentage of ciliated cells ± SD, calculated from an average of 32 images from three biological replicates for human, porcine, and bovine AEC cultures (ns, not significant) **(C)**.

Both ICV and IDV utilize the same receptor determinant to enter the host cell, however both viruses have distinct host reservoirs; human for ICV and bovine for IDV (8, 9, 12, 16–18). Nonetheless, despite the common receptor determinant usage, IDV has shown to have a broader host tropism compared to ICV, suggesting that other factors might play a dominant role in cross-species virus transmission (7). To evaluate this in more detail, we established biologically relevant AEC cultures of human, porcine, and bovine origin as these organisms are reported to be susceptible to either ICV or IDV infection (23, 45–47). To validate the epithelial structure and morphology of the AEC cultures from these host species, we immunostained the fixed inserts for well-established and conserved cellular markers, such as nuclei (DAPI), cilia (β-tubulin), and tight-junctions (ZO-1). This analysis showed that after 4 weeks of differentiation, the AEC cultures of all species had a pseudostratified distribution of the nuclei and presence of a clear tight junction framework along with a homogenous distribution of ciliated cells (**Figures 1A, B**). Because the combined β-tubulin and ZO-1 staining allows for the differentiation between ciliated and non-ciliated cell populations (**Figure 1A**), we quantified microscopy images to determine the percentage of ciliated cells in each of the AEC cultures. This analysis demonstrated that overall porcine AEC cultures have the highest percentage (79 ± 9) of ciliated cells, followed by bovine (71 ± 2) and human (66 ± 3) AEC cultures (**Figure 1C**).

**Figure 2:**
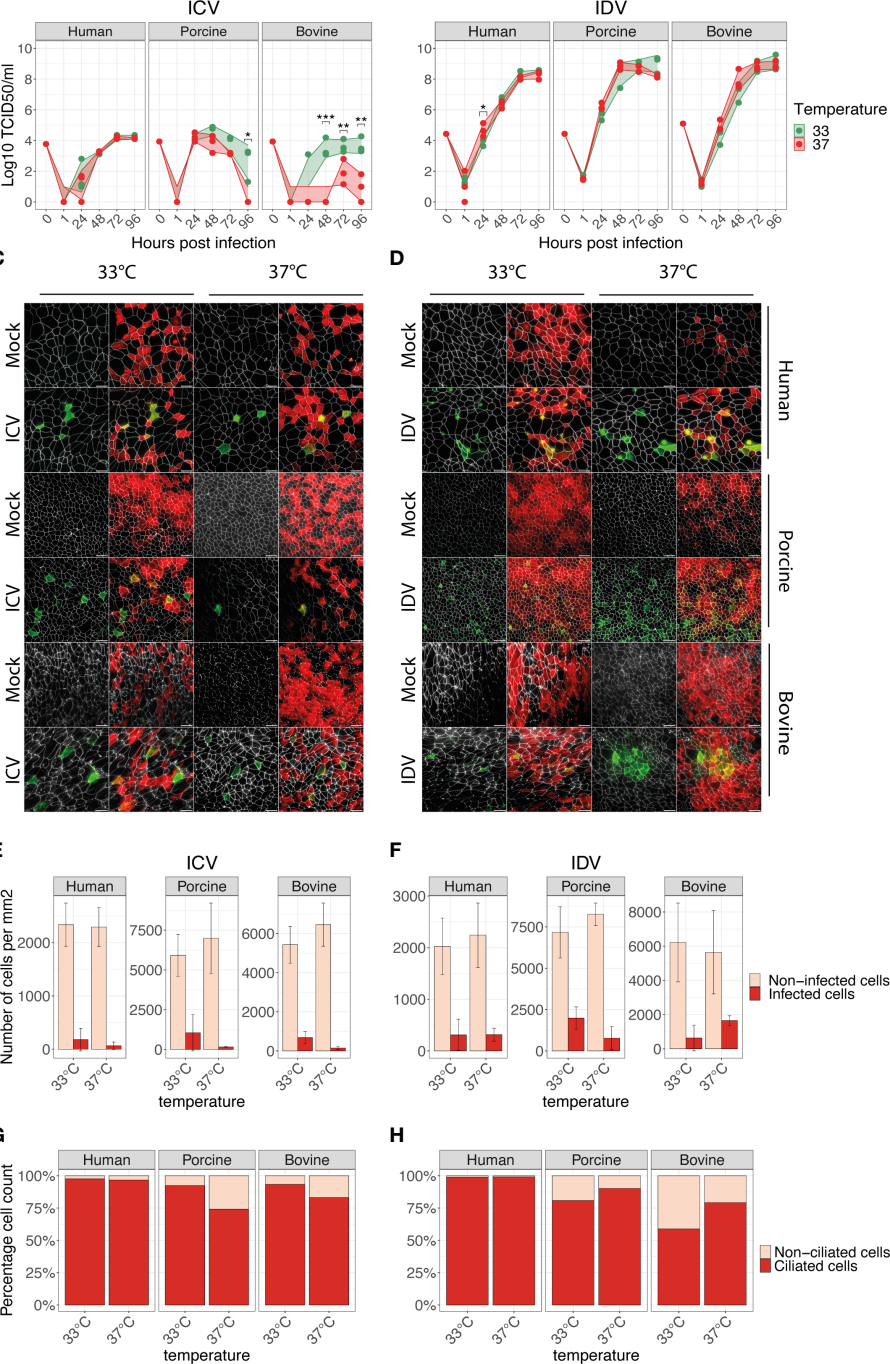
ICV and IDV replication kinetics in human, porcine, and bovine AEC cultures. Well-differentiated AEC cultures from the three different species were infected with ICV **(A)** or IDV **(B)** using 10’000 TCID_50_ or remained uninfected (Mock) and were incubated at 33°C or 37°C. Inoculated virus was removed after 1-hour post-infection (hpi) followed by washing the apical side of the insert. Progeny virus release was assessed by hemagglutination assay at the indicated time post-infection **(A, B)**. Data represent the mean ± SD of AEC cultures from 3 different donors for each species (*p < 0.05, **p < 0.01, ***p < 0.001). Individual biological replicates represent the average of 2 technical replicates. Titers of the inoculum are indicated with 0 hpi, whereas 1 hpi indicates the residual viral titer after the third apical wash. At 96 hpi AEC cultures were formalin-fixed and processed for immunofluorescence analysis using antibodies against ICV (IVIg, green) **(C)** or IDV (anti-NP, green) **(D)**, ZO-1 (tight junctions, white) and β-tubulin (cilia, red). Representative z-projections of control and virus-infected human, porcine, and bovine AEC cultures are shown. Scale bar: 20 µm **(C, D)**. Quantification of ICV **(E)** and IDV **(F)** antigen-positive (dark red) and negative (light red) cells per mm^2^ using automated segmentation of individual cells based on the ZO-1 staining. Data represent the mean ± SD of 48 images acquired per condition (total 576) from 3 different donors for each species **(E, F)**. Determination of ICV **(G)** and IDV **(H)** cell tropism by quantifying the percentage of virus-antigen positive cells overlapping with either ciliated (dark red) or non-ciliated (light red) cells. The mean percentage from all previously quantified virus-antigen positive cells for each temperature and host species is displayed **(G, H)**.

Following the establishment and phenotypical characterization, we employed these well-differentiated AEC cultures from human, porcine, and bovine origin to determine their respective susceptibility to ICV and IDV infection. For this, we inoculated the AEC cultures with 10’000 TCID_50_ of either ICV or IDV and monitored the viral replication kinetics every 24 hours for a total duration of 96 hours. Because we and others previously demonstrated that temperature plays an important role during virus replication, we performed viral replication kinetic experiments at both 33°C and 37°C to assess the influence of the ambient temperature on virus replication efficiency among the different host species (23, 27, 28, 45, 46). As observed in single-species studies, in our multispecies analysis we confirm the ability of both ICV and IDV to replicate in human, porcine, and bovine AEC cultures, with IDV viral titers being 3 to 4 orders of magnitude higher compared to ICV (**Figures 2A, B**). These results are in line with our previous observation in human AEC cultures (23) and indicate that IDV replicates more efficiently compared to ICV in human, porcine, and bovine AEC cultures.

Another noticeable difference between both viruses was the influence of temperature on the viral replication kinetics in the different host species. We reveal that the viral replication kinetics for ICV in human AEC cultures is not detrimentally influenced by temperature. However, in both porcine and bovine AEC cultures, the viral replication kinetics of ICV were severely hampered at 37°C compared to 33°C, as indicated by relatively low viral titers (**Figure 2A**). In contrast, for IDV we observed that irrespective of the host species evaluated, the viral replication kinetics were not severely influenced by temperature, albeit in the porcine AEC cultures IDV titers seemed to remain higher at 33°C compared to 37°C (**Figure 2B**).

To determine if the observed differences between ICV and IDV are influenced by cell tropism, we performed immunofluorescence analysis of virus-infected AEC cultures at 96 hpi (**Figure 2C, D**). For this we quantified the total number of virus-antigen positive and negative cells for 48 randomly selected images for each condition. Our quantification revealed that in all host species, ICV-antigen positive cells per mm^2^ were increased at 33°C compared to 37°C (**Figure 2E**), while for IDV-antigen positive cells no clear temperature-dependent trend was visible (**Figure 2F**). These results are in line with the viral titers observed at 96 hpi (**Figures 2A, B**). Following the quantification of the total number of virus-infected cells, we also determined the respective cell tropism for each virus, and we demonstrate that irrespective of host species and temperature, both ICV and IDV predominantly infect ciliated cells (**Figures 2G, H**).

Combined our multispecies comparison analysis shows that both ICV and IDV can infect human, porcine, and bovine AEC cultures and we quantitatively demonstrate that the respective viral cell tropism does not play a dominant role in the distinctive replication efficiencies of ICV and IDV in AEC cultures from human, porcine, and bovine origin. Because of the effect observed on ICV kinetics with different host species and temperature, other determinants such as cellular-host factors possibly influence the distinct viral replication of ICV and IDV in the different host species.

### Temperature influences the host transcriptional response during ICV and IDV infection

Given the distinctive replication profiles between ICV and IDV in human, porcine, and bovine airway epithelium, we sought to understand to what extent this was influenced by the respective host transcriptional responses. Because of the distinct replication dynamics between ICV and IDV, as well as the potential influence of temperature on the underlying host transcriptional dynamics, we performed a time-resolved transcriptome analysis at 33°C and 37°C. For this we extracted total RNA from cell lysates of ICV- and IDV-infected and non-infected AEC cultures at 24, 48, 72, and 96 hpi. The resulting 216 total RNA samples, 72 per species, were then used as input for BRB-seq and sequenced to a raw sequencing depth of 5 million reads per sample to attain a detail-oriented temporal overview of viral and host gene expression (27).

**Figure 3:**
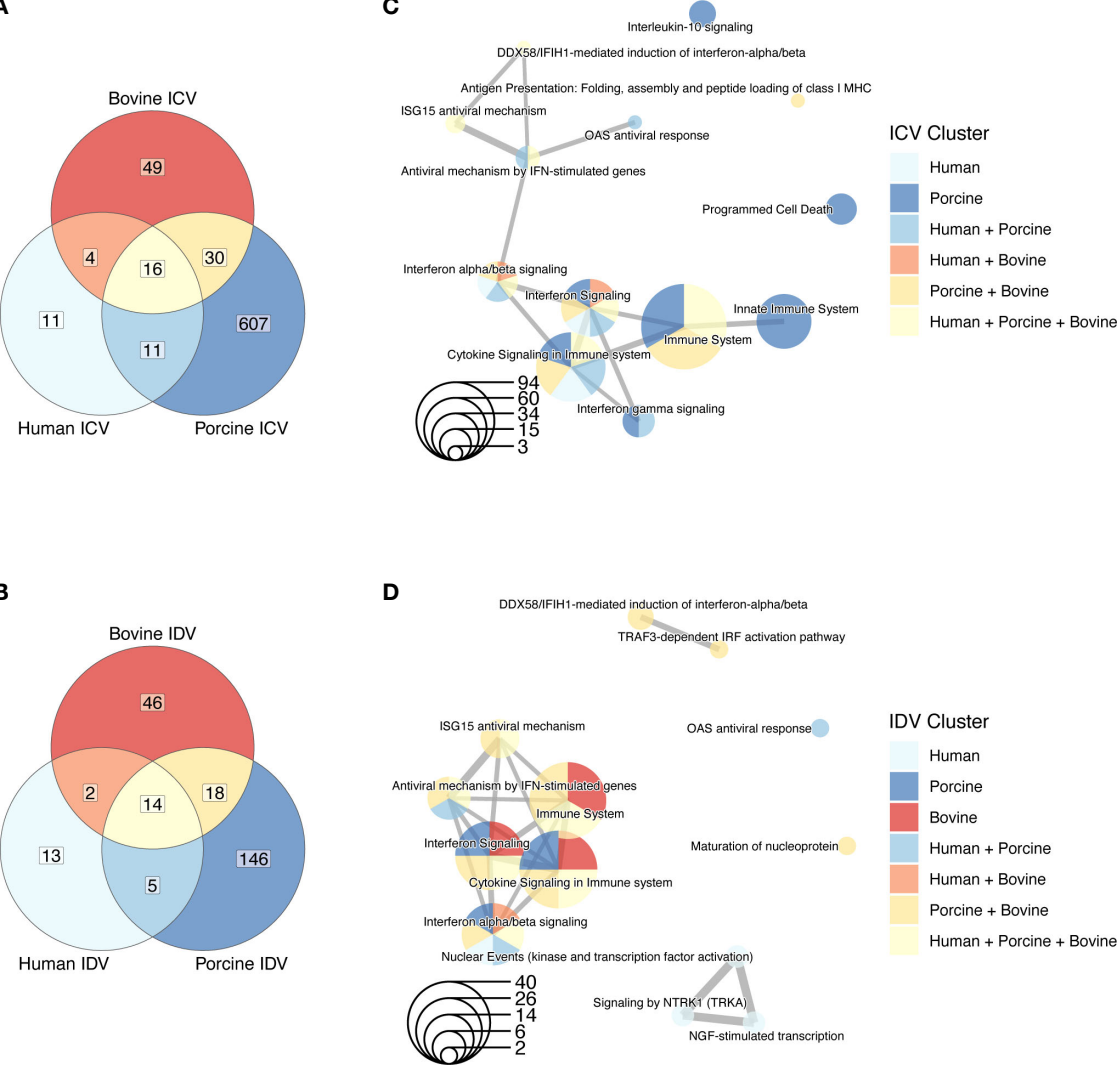
Species-specific DE genes in human, porcine, and bovine AEC cultures during ICV and IDV infection. Venn diagrams of DE genes identified in human, porcine, and bovine AEC cultures during ICV **(A)** and IDV **(B)** infection (FC ≥ 1.5, FDR ≤ 0.05). Enrichment map of connecting networks detected in the gene clusters of the Venn diagrams during ICV **(C)** or IDV **(D)** infection.

In our analysis, we first aimed to identify global differences between ICV and IDV infections among the different species. We used the cumulative normalized data from 3 donors for each species, irrespective of time or temperature, and analyzed the number of DE genes in ICV- and IDV-infected AEC cultures relative to uninfected AEC cultures (FC ≥ 1.5, FDR ≤ 0.05). This revealed that ICV and IDV infections induce a species-specific host response, as the number of common identified DE genes among the different host species is low (**Figures 3A, B** and **Supplementary Table S1**). However, subsequent pathway enrichment analysis (FDR ≤ 0.01) of the different DE gene clusters revealed that despite the little overlap, most genes are associated with innate immune regulatory and response pathways (**Figures 3C, D**). A similar finding emerged when we segregated the data by temperature (**Supplementary Figure S1**, **Supplementary Table S2**). These findings reveal that despite the occurrence of species-specific transcriptomic changes, in all the three host species the major pathway undergoing changes during ICV and IDV infection is the innate immune response pathway.

**Figure 4:**
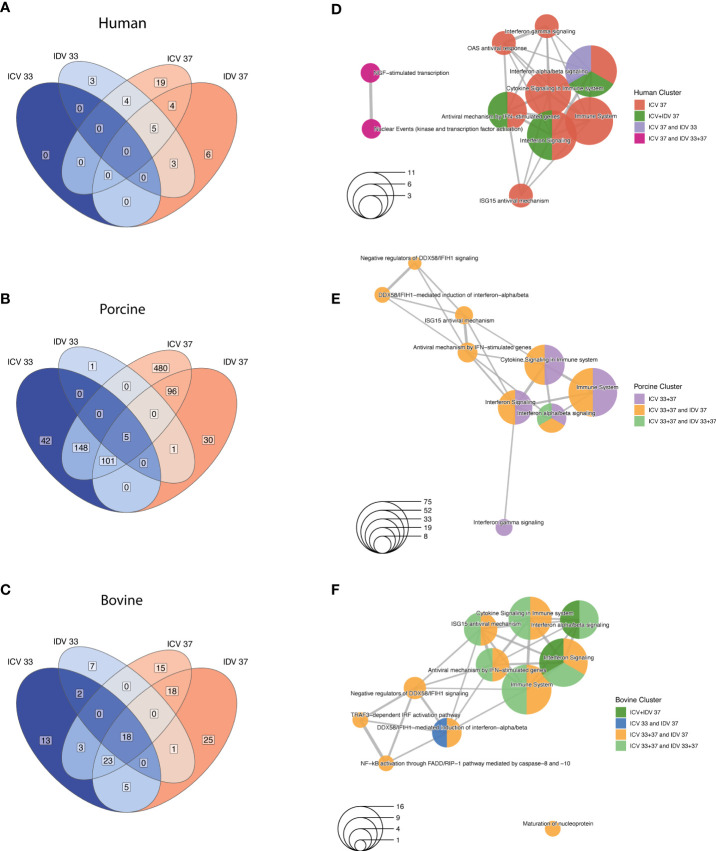
Temperature-dependent analysis of DE genes in human, porcine, and bovine AEC cultures during ICV and IDV infection. Species-specific Venn diagrams of DE genes identified in human **(A)**, porcine **(B)**, and bovine **(C)** AEC cultures during ICV and IDV infection at either 33°C or 37°C (FC ≥ 1.5, FDR ≤ 0.05). Enrichment map of connecting networks detected in human **(D)**, porcine **(E)**, and bovine **(F)** of the aforementioned DE genes identified during ICV and IDV infection in human, porcine, and bovine AEC cultures.

Alongside host species-specific responses, we also investigated common DE genes between ICV- and IDV-infected AEC cultures relative to non-infected AEC cultures at 33°C or 37°C. This demonstrated that DE genes shared between both viruses and temperatures were identified only in porcine (5 DE genes) and bovine (18 DE genes) infected AEC cultures (FC ≥ 1.5, FDR ≤ 0.05) (**Figures 4A–C** and **Supplementary Table S3**). However, pairwise comparison of ICV- and IDV-infected cultures highlighted a possible temperature-dependent trend, with the highest number of DE genes at 37°C compared to 33°C for both ICV- and IDV-infected cultures (**Figures 4A–C**). This trend was most pronounced for ICV-specific DE genes, which increased from 0 and 42 DE genes at 33°C to 19 and 480 DE genes at 37°C in human and porcine ICV-infected AEC cultures, respectively (**Figures 4A, B**). Subsequent pathway enrichment analysis (FDR ≤ 0.01) on the individual DE gene clusters indicated, as observed previously, that both virus-specific (porcine ICV 33 + 37°C and bovine ICV 37°C clusters; **Figures 4E, F**), as well as temperature-dependent (human and bovine ICV+IDV 37°C cluster; (**Figures 4D, F**) DE genes are predominantly associated with pathways related to the innate immune response (**Figures 4D–F**). These results indicate that the host transcriptional response during ICV and IDV infection in human, porcine, and bovine airway epithelial cells is influenced by temperature and is both species- and virus-specific.

### Temporal and temperature-dependent innate immune response dynamics

**Figure 5:**
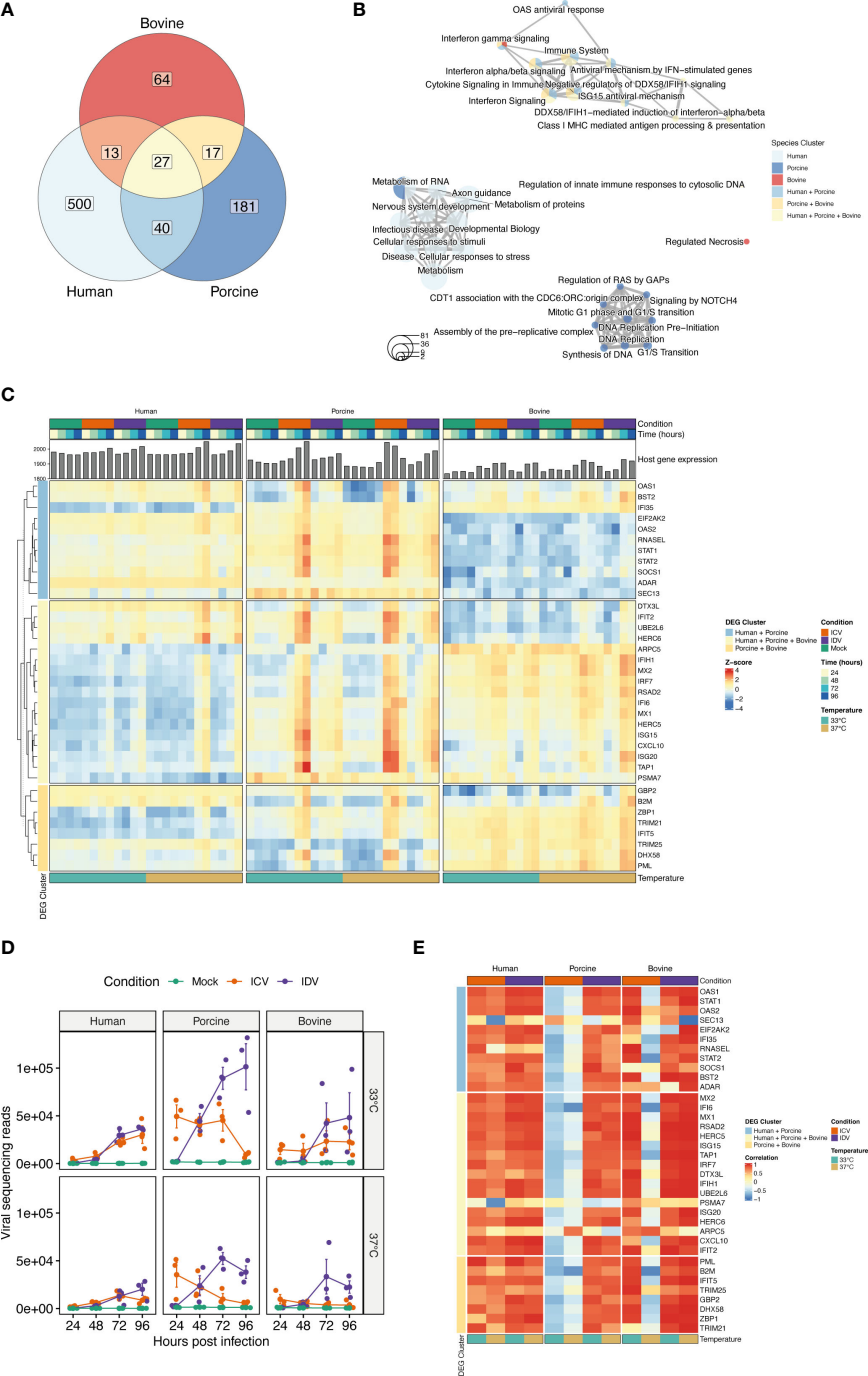
Time-resolved analysis of innate immune response-related DE genes in human, porcine, and bovine AEC cultures during ICV- and IDV infection at 33°C and 37°C. Temporal differences of viral and host gene expression was analyzed from total RNA extracted from cell lysates of ICV and IDV-infected and non-infected AEC cultures at 24, 48, 72, and 96 hpi. Venn diagram displaying the overlap among DE genes identified during ICV and IDV infection in human, porcine, and bovine AEC cultures at 33°C and 37°C (FDR ≤ 0.01) **(A)**. Enrichment map illustrating the functional classification of the aforementioned DE genes identified during ICV and IDV infection in human, porcine, and bovine AEC cultures **(B)**. Heatmap of hierarchical clustered innate immune response-related DE genes subdivided based on their commonality among host species displaying time-resolved changes during ICV (orange) or IDV (purple) infection at 33°C (turquoise) or 37°C (light brown) over the time of infection (gradient of blue) **(C)**. Viral read abundance at the indicated time post-infection **(D)**. Data represent the mean ± SD of AEC cultures from 3 different donors for each species. Correlation of innate immune response-related DE gene expression with viral read abundance in ICV (orange) or IDV-infected AEC cultures (purple) at 33°C (turquoise) or 37°C (light brown) **(E)**.

Because the previous global analysis approach is not tailored to identify genes that are dynamically regulated across the different time points, temperatures, and experimental conditions, we performed a Likelihood ratio test (LRT) for each individual host species to detect genes that change significantly during virus infection at 33°C and 37°C. This approach identified a total of 696, 367, and 199 DE genes for human, swine, and bovine, respectively (FDR ≤ 0.01) (**Supplementary Table S4**). To identify groups of DE genes with similar expression profiles, we performed hierarchical clustering analysis and identified a total of 580, 265 and 121 DE genes for human, swine, and bovine hosts, respectively (**Figure 5A**). When we assessed the overlap of these genes between the different species, we found that most of the DE genes are species-specific and that, similarly to the global analysis approach, only a small fraction of DE genes is shared between species (**Figure 5A** and **Supplementary Table S5**). In total, 27 DE genes are common among all species, 40 are shared between human and porcine, and 13 and 17 are common among the human-bovine and porcine-bovine pairs, respectively (**Figure 5A**). Functional classification of the different DE gene clusters showed that the species-specific DE gene clusters are associated with cell metabolism or DNA replication/synthesis (**Figure 5B**). In contrast, most of DE genes common to at least two or all host species were associated with pathways involved in the innate immune response, such as interferon (IFN) and cytokine signaling, as well as IFN-dependent antiviral responses (**Figure 5B**).

Closer inspection of the DE gene expression level dynamics revealed that only the genes common to two or all host species displayed a clear temporal and temperature-dependent expression pattern (**Figure 5C** and **Supplementary Figure S2**, **Supplementary Table S5**). This includes interferon-stimulated genes (ISGs) that are involved in antigen presentation (TAP1, PSMA7, B2M), transcription of interferons and pathogen recognition (IRF7, IFIH1/MDA5), as well as the host antiviral response (CXCL10, IFIT2, ISG15, ISG20, MX1, MX2, RSAD2/Viperin, IFI6) (**Figure 5C**) (48–51). These identified genes displayed a virus-specific expression profile among the different host species, with enhanced expression levels at 37°C compared to 33°C (**Figure 5C**). ISGs involved in the OAS/RNaseL pathway as well as the ISG-related transcriptional regulator genes STAT1, STAT2, and SOCS1 were shown to be dynamically regulated over time in human and porcine virus-infected AEC cultures (**Figure 5C**) (52). In line with this, we identified GBP2, B2M, DHX58, IFIT5, and PML, as well as both TRIM21 and TRIM25, which are members of the tripartite motif family of E3 Ubiquitin ligases that bidirectionally regulate the intracellular antiviral response, to be only associated with the porcine-bovine cluster (53–55) (**Figure 5C**). Combined these results indicate that the host innate immune response during viral infection is temperature-dependent and differentially regulated over time in a virus and species-specific manner.

Because our sequencing approach also allows obtaining a temporal overview of viral gene expression, we assessed how the respective ICV- and IDV-specific read abundance correlates with the host transcriptional response (27). For this we first examined the viral read abundance for both ICV and IDV, and demonstrate that expression profiles are consistent with the respective progeny viral titers recorded previously (**Figures 2A, B**, **5D**), with both ICV-viral titers and viral read abundance showing more attenuation at 37°C compared to 33°C in both porcine and bovine AEC cultures. Interestingly, in case of IDV the viral read abundance was slightly higher at 33°C compared to 37°C, albeit until 96 hpi this difference did not translate itself in higher viral titers (**Figures 2A, B**, **5D**). Following the inspection of the viral read abundance, we performed correlation analysis to determine if there is a relationship between the viral read abundance of ICV and IDV and certain identified DE genes associated with the host innate immune response. This revealed that during ICV infection there is a pronounced inverse correlation in both porcine and bovine AEC cultures at 37°C, as well as at 33°C in the porcine AEC cultures (**Figure 5E**). In contrast, in ICV-infected human AEC cultures the correlation was positive. In the case of IDV-infected AEC cultures we observed a positive correlation between IDV read abundance and most of the DE genes for all host species, and this correlation seems to be more abundant at 37°C compared to 33°C (**Figure 5E**).

Combined these results demonstrate that there is a dynamic interplay between virus and host in the respiratory epithelium from human, porcine, and bovine origin that is influenced in a temperature-dependent manner. Moreover, our multispecies time-resolved analysis revealed both species-specific and species uniform determinants related to the innate immune pathway, some of which inversely correlated with ICV reads abundance and could possibly play a role in the restricted replication of ICV in swine and cattle. The identification of these host genes warrants further detailed molecular characterization on their respective role in the distinct viral replication efficiencies and host tropism between the human-associated ICV and the ruminant-associated IDV.

## Discussion

To our knowledge this is the first study providing a comprehensive time-resolved comparative analysis of two closely related respiratory viruses that have distinct reservoir hosts, namely human for ICV and cattle for IDV. We used biologically relevant *in vitro* models of the respiratory epithelium of human, porcine, and bovine origin and monitored the viral replication kinetics, host, and cell tropism during ICV- and IDV-infection at different ambient temperatures (33°C and 37°C). This detailed analysis revealed that both ICV and IDV predominantly infect ciliated cells, independently from host and temperature (**Figures 2G, H**). However, despite the shared cell tropism, temperature was shown to have a profound influence on ICV replication in both porcine and bovine AEC cultures, while IDV efficiently replicated in all species irrespective of temperature (**Figures 2A, B**). Detailed time-resolved transcriptome analysis revealed both species-specific and species uniform host responses and highlighted 34 innate immune-related genes with clear virus-specific and temperature-dependent profiles (**Figure 5C**). We show that in porcine (33°C and 37°C) and bovine (37°C) AEC cultures the amplitude of DE genes inversely correlates with the replication efficiency of ICV, whereas in all other cases we observed a positive correlation with ICV and IDV replication efficiency (**Figure 5E**). With these results, we provide first comprehensive insights on important common and species-specific virus-host dynamics in the respiratory epithelium of human, swine, and cattle, as well as highlighting a set of innate immune-related genes as possible key host determinants underlying the distinct host tropism of ICV and IDV.

Receptor recognition is one of the key viral determinants influencing the host range of a virus. It was previously shown that the HEF glycoprotein of ICV and IDV, which both utilize 9-O-SA, binds to the epithelial surface of human, porcine, and bovine respiratory tract and that the more open receptor cavity of IDV-HEF is a possible determinant of the distinct host tropism of ICV and IDV (7). With the use of biologically relevant *in vitro* models, we now further demonstrate that both ICV and IDV can productively infect ciliated cells in respiratory epithelium of human, porcine, and bovine origin (**Figures 2A, B**). Although viral entry is one of the first critical steps to infection, the innate immune response is the first line of defense of the host and one of the key barriers present in all vertebrates to restrict viral infection that further define the infection outcome in the new host (50, 56). Our results suggest that temperature and host species responses might play a profound role in the distinct host range observed for ICV and IDV. However, it cannot be formally excluded that the reported higher binding affinity and the open receptor-binding cavity of the IDV HEF glycoprotein influences the observed difference in replication efficiency for ICV and IDV, nor the observed delayed host innate immune response during IDV compared to ICV infection. Furthermore, as detailed information regarding the interactions between cellular co-factors with the viral RNA polymerase complexes of ICV and IDV is missing, it cannot be excluded that in certain host species the efficiency of such interactions is affected, nor whether this may lead to enhanced pathogen recognition receptor sensing, as described for other influenza viruses (57). Therefore, further molecular characterization of viral determinants using the available reverse genetic systems for ICV and IDV in combination with the described *in vitro* models will shed more light on their respective roles in cross-species transmission (58, 59).

In our study, we demonstrate that ambient temperatures reminiscent of the human upper and lower respiratory tract, 33°C and 37°C respectively, can influence both the viral replication efficiency and the amplitude of the cell-intrinsic innate immune response in different host species. These temperatures were selected, as detailed thermal mapping of the respiratory tract temperatures of porcine and bovine has not yet been described and core rectal measurement remains the preferential method for assessment of the core body temperature for both porcine and bovine (38 – 40°C and 38 – 39°C, respectively) (60, 61). However, given that both livestock animals utilize respiration to thermoregulate their core body temperatures (60, 62, 63), we hypothesize that the temperature in the respiratory tract should be cooler respective to the core body temperature. Nonetheless, as fever is an evolutionary conserved response to viral and bacterial infections (64, 65), it would be of interest to assess the viral replication efficiency and host response dynamics at hyperthermic temperatures (39 – 41°C) during ICV and IDV infection in different host species. Especially as, in contrast to lower ambient temperatures, viral replication in human airway epithelial cells has previously been described to be restricted at high ambient temperatures independently of the host IFN-mediated antiviral immune response (66). Therefore, given the economic importance of pigs and cows and their role as a host reservoir of potential emerging diseases, detailed thermal mapping at different anatomical regions in the respiratory tract, as well as determining the influence of hyperthermic temperatures on ICV and IDV replication efficiency and cross species transmission among these host species remain warranted.

Our time-resolved transcriptome analysis among the three host species uncovered that the host innate immune response during ICV and IDV infection is temperature-dependent and differentially regulated over time in a virus-specific manner, as reflected by the poor replication of ICV in porcine and bovine AEC cultures. Moreover, our results also demonstrate that the repertoire of DE genes expressed during virus infection in different host species includes both common and species-specific genes. These results are in line with the study by Shaw and colleagues, who investigated the transcriptional host response in IFN-stimulated primary fibroblasts from 10 vertebrate species (50). Interestingly, 17 of the 34 DE genes identified in our study overlap with the 62 core ISGs that have previously been identified in the study by Shaw and colleagues (50). The modest overlap in the DE genes between both studies is likely due to the intrinsic difference in the experimental setup and *in vitro* models used. Nevertheless, our results demonstrate that the host response is also conserved in more biological relevant *in vitro* models with distinctive heterologous cell populations. Whether the host response is conserved among individual cell populations from different host species, as well as in virus-infected and uninfected cells remains to be determined.

Since a large fraction of the identified DE genes that are dynamically regulated during ICV and IDV infection were previously shown to be involved in antiviral responses during influenza A (IAV) infection (48, 49), our results suggest that despite the distant phylogenetical relationships and the distinct receptor usage between the different genera, certain aspects of virus biology seem to be conserved. Our detail-oriented transcriptome analysis also uncovered that the amplitude of DE gene expression can directly be correlated with the viral replication efficiency in a virus- and species-specific manner that is influenced by temperature. These results indicate that successful interspecies transmission is temperature-dependent, but foremost it relies on the conservation of virus-host interactions, such as the ability for a virus to antagonize the IFN response in multiple host species. Because in ICV-infected porcine and bovine AEC cultures we observed an inverse correlation between viral and host gene expression, this suggests a decreased ability of ICV to antagonize the antiviral responses of these host species. In this context, DE genes common to only porcine and bovine AEC cultures, such as GBP2, B2M, DHX58, IFIT5, and PML, TRIM21, and TRIM25, could act as important restriction factors. Therefore, further detailed molecular characterization of the interaction between ICV and IDV and individual orthologous host determinants will be important to elucidate the role of the respective 34 identified DE genes in the multifaceted intricated nature of cross-species transmission.

Combined, we reveal that the reaction of the innate immune response of the airway epithelium is dependent on many variables, such as time, temperature, host species, and pathogen. Taking into account all these conditions renders conclusion-making challenging, but it provides more detailed and representative information about the high complexity of the innate immune response towards infection, an extremely important aspect in the context of emerging diseases and zoonoses. Because we are lacking knowledge on the dynamics of the innate immune response in host species other than human and mouse, with our multispecies study we aimed to obtain more information about the innate immune response of species relevant to the host range of both ICV and IDV. In addition to this, we provide a list of possible determinants involved in viral cross-species transmission that deserve closer functional and molecular characterization.

## Data availability statement

Transcriptome data has been deposited in the Arrayexpress open-access public repository from the European Bioinformatics Institute (EMBL-EBI) under E-MTAB-11939.

## Ethics statement

This study was reviewed and approved by the ethics commission Canton St. Gallen; EKSG 11/044, EKSG 11/103 and the ethics commission Canton Bern: KEK-BE 302/2015 and KEK-BE 1571/2019. The patients/participants provided their written informed consent to participate in this study. The animal study was reviewed and approved by Amt für Landwirtschaft und Natur LANAT, Veterinärdienst VeD, Bern, Switzerland, with the agreements BE87/17 and BE92/20.

## Author contributions

RD designed the study. LL performed data analysis and experiments. LL and RD performed data analysis and manuscript preparation. MFL, LP, ML, MG, PV, and MH helped in performing data experiments, data analysis and manuscript preparation. All authors contributed to the article and approved the submitted version.

## Funding

This research was funded by the Swiss National Science Foundation, grant number 310030_179260.

## Acknowledgments

We would like to thank Georg Herrler, University of Veterinary Medicine Hannover, Germany, for providing ICV (C/Johannesburg/1/66) and Feng Li, South Dakota University, United States, for providing IDV (D/bovine/Oklahoma/660/2013). We also would like to thank Nicolas Ruggli and the team of animal caretakers at the Institute of Virology and Immunology, Mittelhäusern, Switzerland, for their assistance in providing chicken blood. Furthermore, we are grateful to Sabina Berezowska and Irene Ramos-Centeno (Institute of Pathology, University of Bern) for providing the respiratory tract tissues via the Tissue Bank Bern.

## Conflict of interest

The authors declare that the research was conducted in the absence of any commercial or financial relationships that could be construed as a potential conflict of interest.

## Publisher’s note

All claims expressed in this article are solely those of the authors and do not necessarily represent those of their affiliated organizations, or those of the publisher, the editors and the reviewers. Any product that may be evaluated in this article, or claim that may be made by its manufacturer, is not guaranteed or endorsed by the publisher.

## Supplementary material

Supplementary Figure 1 Virus-specific Venn diagrams of DE genes identified in human, porcine, and bovine AEC cultures during either ICV **(A, B)** or IDV **(C, D)** infection at 33°C **(A, C)** or 37°C **(B, D)**, respectively. Enrichment map of connecting networks detected of the aforementioned DE genes identified during ICV **(E, F)** or IDV infection **(G, H)** at 33°C **(E, G)** or 37°C **(F, H)**, respectively.

Supplementary Figure 2 Heatmap of hierarchical clustered of all DE genes subdivided into the aforementioned DE gene clusters identified during ICV (orange) or IDV (purple) infection at 33°C (turquoise) or 37°C (light brown) over the time of infection (gradient of blue).

Supplementary Table S1 DE genes identified during global analysis during either ICV or IDV infection among the different species (FC ≥ 1.5, FDR ≤ 0.05).

Supplementary Table S2 DE genes identified during global analysis during either ICV or IDV infection among the different species during either 33°C or 37°C (FC ≥ 1.5, FDR ≤ 0.01).

Supplementary Table S3 DE genes identified during global analysis during both ICV and IDV infection in human, porcine, and bovine AEC cultures (FC ≥ 1.5, FDR ≤ 0.05).

Supplementary Table S4 DE genes identified for each individual host-species during ICV and IDV infection (Likelihood ratio test (LRT) , FDR ≤ 0.01).

Supplementary Table S5 DE genes identified by hierarchical clustering analysis for human, porcine, and bovine AEC cultures during ICV and IDV infection corresponding to **Figure 5A** and **Supplementary Figure S2** and **Figure 5C**.
