# Supplementary figures and images for: Time-resolved characterization of the innate immune response in the respiratory epithelium of human, porcine, and bovine during influenza virus infection
Source: Front Immunol. 2022 Aug 19;13:970325. doi: 10.3389/fimmu.2022.970325 (PMC9437644; doi:10.3389/fimmu.2022.970325)

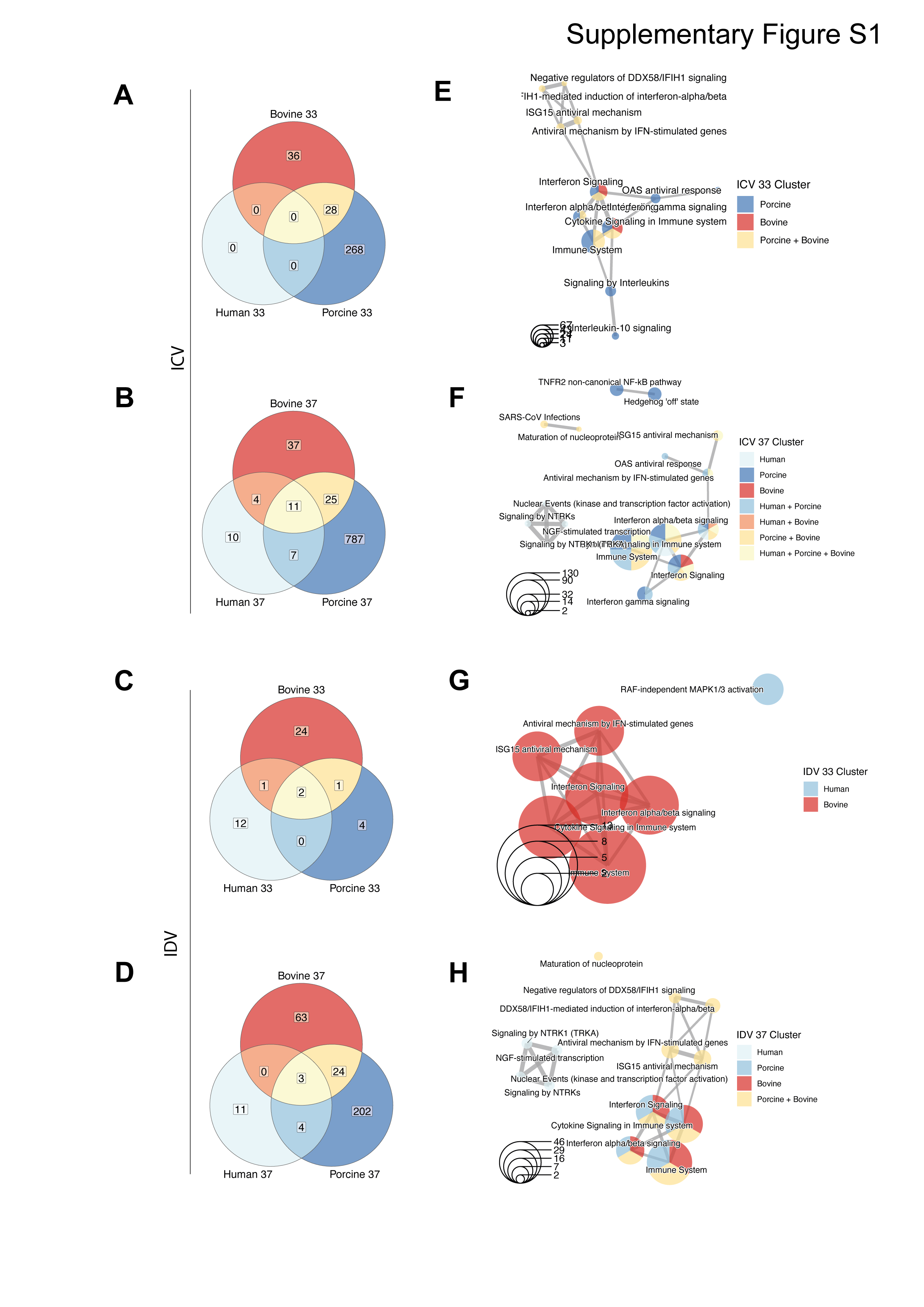

Supplement: Supplementary Figure 1 — Virus-specific Venn diagrams of DE genes identified in human, porcine, and bovine AEC cultures during either ICV (A, B) or IDV (C, D) infection at 33°C (A, C) or 37°C (B, D), respectively. Enrichment map of connecting networks detected of the aforementioned DE genes identified during ICV (E, F) or IDV infection (G, H) at 33°C (E, G) or 37°C (F, H), respectively. [file Image_1.tif]

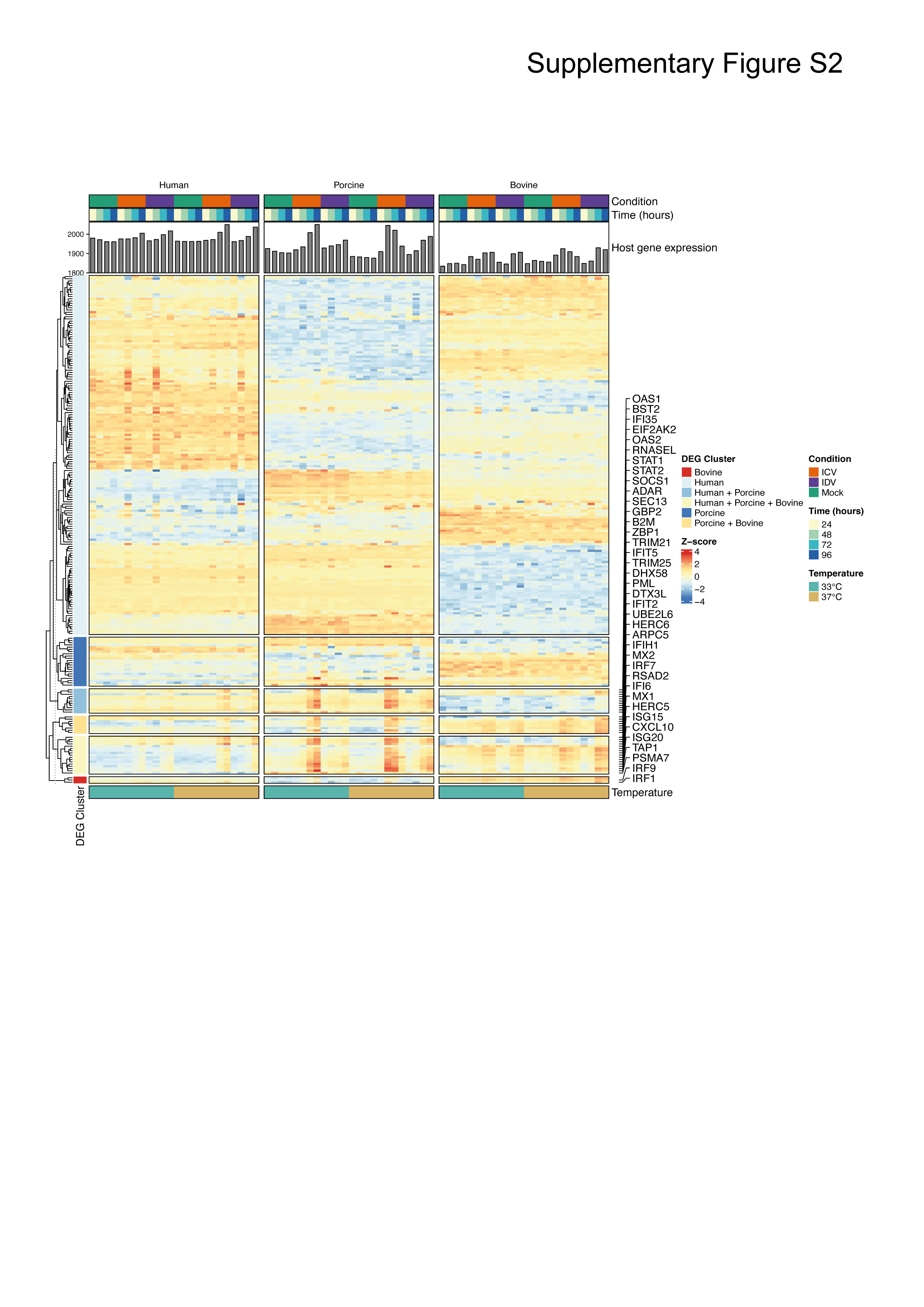

Supplement: Supplementary Figure 2 — Heatmap of hierarchical clustered of all DE genes subdivided into the aforementioned DE gene clusters identified during ICV (orange) or IDV (purple) infection at 33°C (turquoise) or 37°C (light brown) over the time of infection (gradient of blue). [file Image_2.tif]
